# Heredity, Health, and Personal Beauty

**Published:** 1891-06

**Authors:** 


*Heredity, Health, and Personal Beauty.* By JOHN V. SHOEMAKER, M.D. Pp. 422. Philadelphia: F. A. DAVIS. 1890.

The mode of exchanging ideas which is known amongst us as conversation is represented under certain circumstances by Americans as "Talk," and in its most advanced development as "Tall Talk." This is some "Tall Talk" by a gentleman whose chief pursuit, amongst many described on the title-page, would appear to be Dermatology. From Darwin and evolution to pimples, and cunning concoctions for the cure of these, the book treats "*de omnibus rebus et quibusdam aliis.*" Chapter X. is entitled "Grace the Crown of Beauty," and Chapter XXXVI. is headed "List of Medicated Soaps." In this extensive *répertoire* there must be a corner for the special product of the Continent; and, sure enough, Chapter XII. is on "The Evolution of the American Girl"!

We have read the book from beginning to end with astonishment, almost with awe. It is brimful of noble sentiments and elevating advice; it bristles with figures of speech, and is not devoid of distinct efforts at logical reasoning. But somehow we cannot help smiling as we read; for bathos jumbles pathos, the ridiculous ousts the sublime, the culture of the schoolboy handles the thoughts of the sage or the historian, and through the whole peeps the clinical insight of the skin doctor. It is, in fact, a sort of apotheosis of the somewhat unsavoury subject of Diseases of the Skin.

On one beloved, if somewhat tender and delicate, theme he insists, and occasionally waxes eloquent, suspiciously protesting too much; and this is, the pervading cleanly habits of the American, as compared with the less clean (not actually filthy) habits of the Englishman. Here our own mouths are shut; but we may quote from Julien Gordon, one of their own writers, not unknown amongst us, who says: "The American . . . is lamentably untidy—I hesitate to say unclean, but I do not hesitate to assert, and to assert it vehemently, that the first clause is true of our men of all classes, stations, vocations, and degrees of wealth!" Unsavoury details follow. As to defending ourselves, we may safely leave that to the fair American hostess who, when asked why American girls preferred English men, replied, "Oh, their men are so clean!" If his womenfolk are for us on this ground, there is clearly a large field for the skin-specialist among Dr. Shoemaker's male compatriots.

